# Quaternary Knot Technique: Suture Knot Burial without Scleral Flap or Incision for Trans-scleral Fixation

**DOI:** 10.18502/jovr.v18i3.13784

**Published:** 2023-07-28

**Authors:** Levent Dogan, Ibrahim Edhem Yilmaz

**Affiliations:** ^1^Tatvan State Hospital, Department of Ophthalmology, Bitlis, Turkey; ^2^Kilis State Hospital, Department of Ophthalmology, Kilis, Turkey

**Keywords:** Conjunctival Erosion, Endophthalmitis, Knot Burial, Scleral Fixation

## Abstract

Despite the introduction of novel sutureless posterior chamber intraocular lens (IOL) fixation techniques, some conditions still require suture-assisted scleral fixation. If the scleral fixation suture knot is left directly under the conjunctiva, it may become exposed, resulting in an increased risk of endophthalmitis. To avoid this problem, we offer a new alternative, simple, and safe way for burying the end of the suture using knots in this report.

##  INTRODUCTION

In recent years, different advanced techniques, including sutureless posterior chamber intraocular lens (IOL) fixation, have been introduced to be applied in the absence of adequate capsular support.^[1,2,3,4,5]^ On the other hand, suture-assisted fixation may still be necessary in the implantation of a scleral-sutured capsular tension ring (CTR) and iridodialysis repair, as well as in the presence of potential problems associated with sutureless procedures in IOL implantation. Leaving suture knot ends directly under the conjunctiva after scleral suture fixation often results in suture erosion, causing ocular discomfort and an increased risk of endophthalmitis. The scleral flap technique, which is invasive and time consuming, is the most used procedure in the prevention of problems based on suture knot exposure.

In this report, we present a new knot burial technique without the use of a scleral flap or incision.

**Figure 1:**
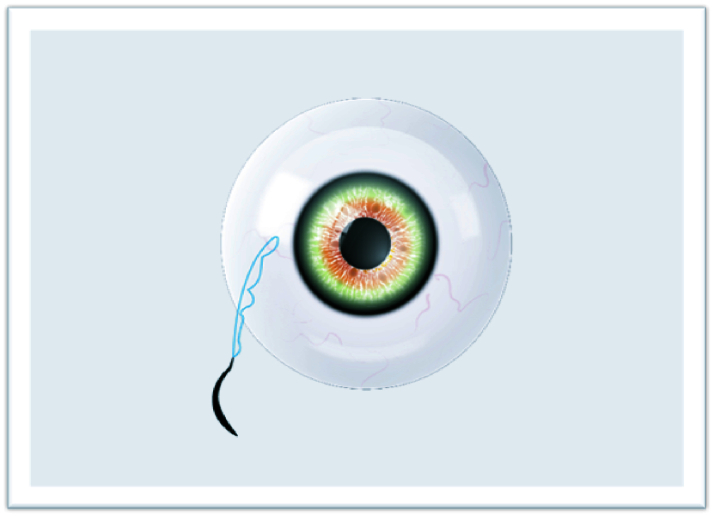
Externalized suture.

**Figure 2:**
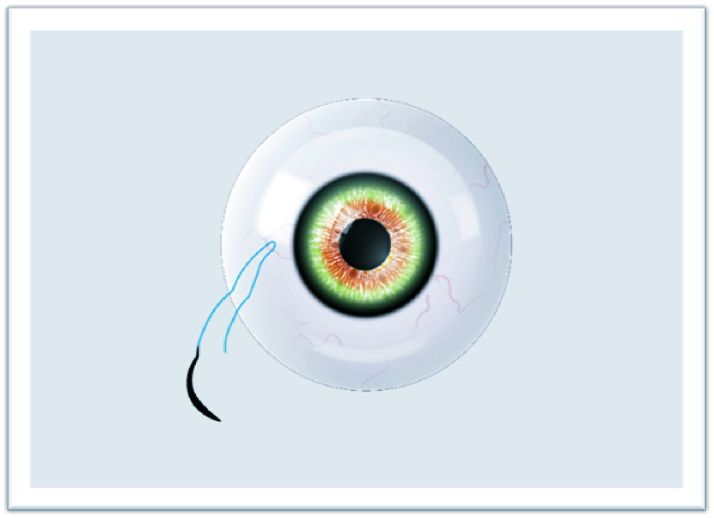
Two separate fibers formed for suturing.

**Figure 3:**
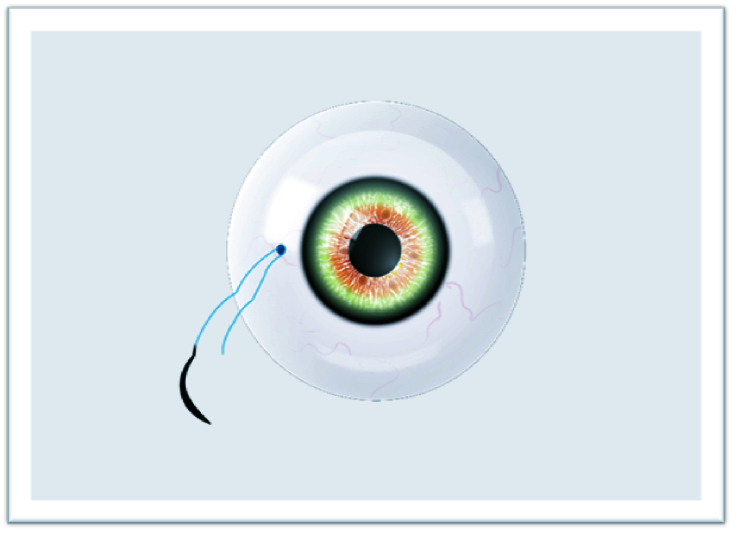
First knot.

**Figure 4:**
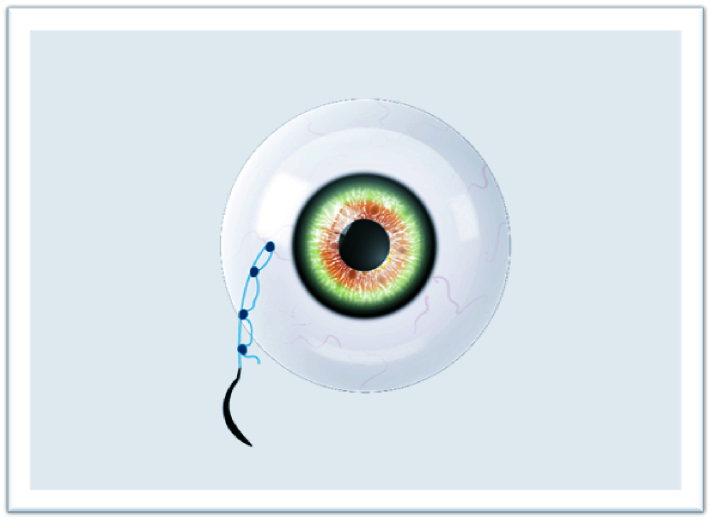
Other knots required for the “quaternary knot” technique.

**Figure 5:**
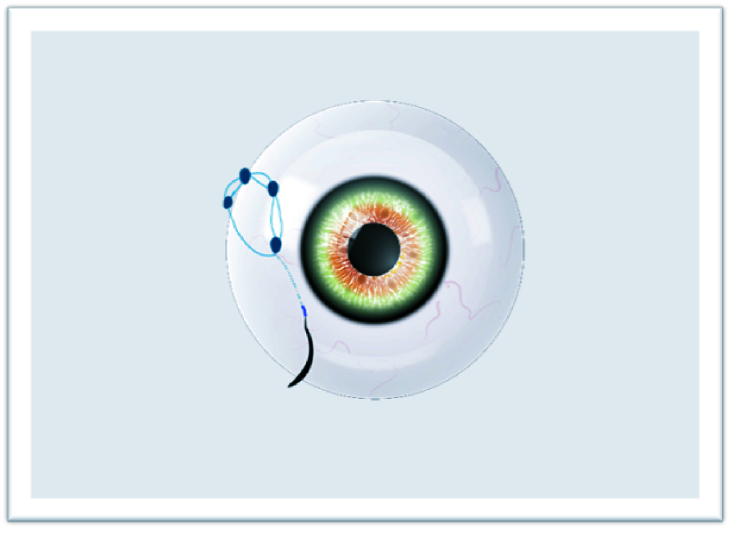
Pulled out needle after scleral lamellar advancing.

**Figure 6:**
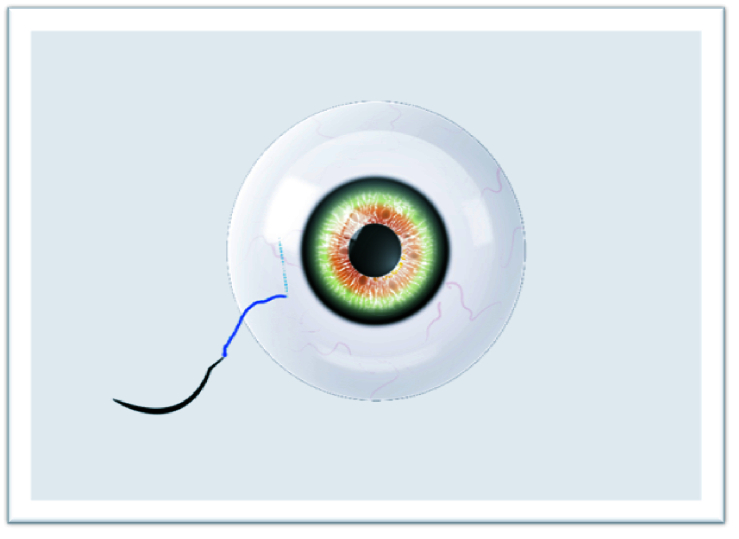
Buried knots after fully externalized suturing.

**Figure 7:**
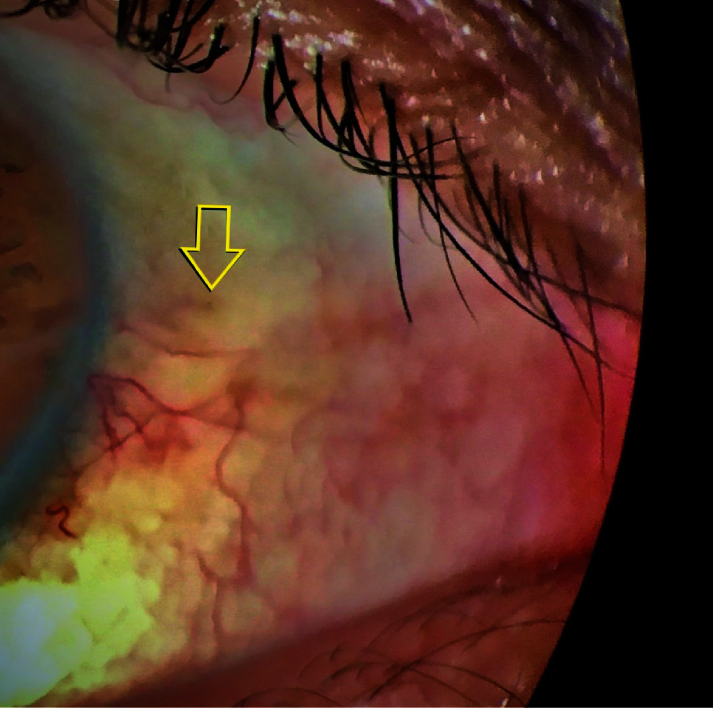
Postoperative third-month appearance of buried knots (only a little part of the first knot is seen as indicated by a yellow arrow, and there is no conjunctival erosion).

##  SURGICAL METHOD

The study adhered to the tenets of the Declaration of Helsinki, and written informed consent was obtained from each patient participating in the study. The ethical approval was obtained from the Ethics Committee of Van Training and Research Hospital, Turkey (2215/2021). In this technique, surgery can be performed under retrobulbar or sub-tenon anesthesia. The IOLs or CTRs are attached with a 9-0 looped polypropylene suture (Mani, Inc, model: 2452L, Japan) by passing through the eyelet of implants subsequently from inside the suture loop. The nasal and temporal conjunctiva are sufficiently opened by the surgeon to create conjunctival peritomies. Following that, an anterior vitrectomy is performed with a 20-gauge or 23-gauge vitrectomy cutter and triamcinolone. Then, corneal or scleral incisions are made at a length that lets the implants to pass through. Curved needles are inserted through the primary corneal or scleral incision and bilaterally externalized 2 mm posterior to the limbus [Figure 1]. The IOL or CTR is implanted and manipulated using the Sinskey hook to ensure centralization. One of the fibers of the closed-loop suture is cut approximately 1.5 cm from the exit point of the needles so that two separate fibers are formed for suturing [Figure 2]. Following the suturing of the corneal or scleral incision, the first 3-2-2 knot is produced on the scleral surface to provide a stable scleral fixation [Figure 3]. The second knot is produced solely in a three-way fashion and tied approximately 2 mm away from the first scleral knot. For the “quaternary knot” technique, the third and fourth knots are formed using the same procedure in the second knot and tied at 2 mm intervals [Figure 4]. After the fourth knot, the free suture end (without a needle) is cut away from where the last knot terminates, resulting in a single suture with a scleral knot and three knots on the suture. The needle is inserted into the sclera at the point as close as possible to the first knot and advanced about 5 mm into the sclera in a lamellar fashion [Figure 5]. The needle is pulled out, and the knots are buried in the sclera [Figure 6]. Finally, the suture is cut at the exit point. All aforementioned procedures are carried out on the other side. The conjunctiva is reattached securely with 8-0 absorbable suture, and gentamycin-dexamethasone is injected subconjunctivally.

##  RESULTS

We applied this knot-burying technique on a total of 21 patients (two for CTR fixation and one for iridodialysis repair) and did not encounter any problems over an average six-month follow-up period.

##  DISCUSSION

Complications such as IOL dislocation and haptic fracture can occur, and there may be insufficient equipment for sutureless IOL implantation; as a result, surgeons must maintain their transscleral suture-mediated fixing capabilities. Transscleral suturing procedures are also required for scleral-sutured CTRs, iridodialysis repair, and the centralization of previously dislocated one-piece IOLs.

Compared to the scleral flap and Z-suturing procedures, our technique is less invasive, and needs less non-conjunctival area, and also the sclera is less manipulated.^[6]^ The scleral lamellar passage occurs just once after the needle is removed from the sclera for the first time, and no scleral flap or incision is used; hence, less irritation and inflammation are expected.

Suture erosion can lead to ocular discomfort and more serious problems, such as endophthalmitis. To prevent these complications, scleral flaps, autologous cornea patches, dura mater, and fascia lata can be used.^[7,8]^ In our technique, the intermittently buried knots minimize the risk of slippage and suture exposure [Figure 7].

There are some other knot burial methods, such as Baykara's direct suture burial technique and the friction knot technique.^[9,10]^ We consider that our technique might be an alternative to current methods, particularly in terms of preventing slippage by using the buried knot technique.

##  Financial Support and Sponsorship

None.

##  Conflicts of Interest 

None.
